# A comparison of the prevalence of prenatal alcohol exposure obtained via maternal self-reports versus meconium testing: a systematic literature review and meta-analysis

**DOI:** 10.1186/1471-2393-14-127

**Published:** 2014-04-03

**Authors:** Shannon Lange, Kevin Shield, Gideon Koren, Jürgen Rehm, Svetlana Popova

**Affiliations:** 1 Social and Epidemiological Research Department, Centre for Addiction and Mental Health, 33 Russell St, M5S 2S1 Toronto, ON, Canada; 2 Dalla Lana School of Public Health, University of Toronto, 155 College St, M5T 3M7 Toronto, ON, Canada; 3 Institute of Medical Science, University of Toronto, 1 King’s College Cir, M5S 1A8 Toronto, ON, Canada; 4 Division of Children’s Health and Therapeutics, Children’s Health Research Institute, 800 Commissioners Rd. E, N6C 2V5 London, ON, Canada; 5 Departments of Medicine, Paediatrics, Physiology & Pharmacology, Schulich School of Medicine & Dentistry, Western University, 1151 Richmond St, N6A 5C2 London, ON, Canada; 6 Ivey Chair in Molecular Toxicology, Western University, 1151 Richmond St, N6A 5C2 London, ON, Canada; 7 The Motherisk Program, The Hospital for Sick Children, 555 University Ave, M5G 1X8 Toronto, ON, Canada; 8 Departments of Paediatrics, Pharmacology, Pharmacy and Medical Genetics, University of Toronto, 1 King’s College Cir, M5S 1A8 Toronto, ON, Canada; 9 Epidemiological Research Unit, Klinische Psychologie & Psychotherapie, Technische Universität Dresden, Chemnitzer Str. 46, D-01187 Dresden, Germany; 10 Factor-Inwentash Faculty of Social Work, University of Toronto, 246 Bloor St. W, M5S 1V4 Toronto, ON, Canada

**Keywords:** Biomarkers, Meconium, Prenatal alcohol exposure, Prevalence, Maternal self-reports

## Abstract

**Background:**

Maternal self-reports, used for the detection of prenatal alcohol exposure (PAE), may lack validity, necessitating the use of an objective biomarker. The detection of fatty acid ethyl esters (products of non-oxidative ethanol metabolism) in meconium has been established as a novel biomarker of PAE. The purpose of the current study was to compare the prevalence of PAE as reported via maternal self-reports with the results of meconium testing, and to quantify the disparity between these two methods.

**Methods:**

A systematic literature search for studies reporting on the prevalence of PAE, using maternal self-reports in combination with meconium testing, was conducted using multiple electronic bibliographic databases. Pooled prevalence estimates and 95% confidence intervals (CI) were calculated based on eight studies, using the Mantel-Haenszel method, assuming a random effects model. A random effects meta-regression was performed to test for a difference.

**Results:**

The pooled prevalence of PAE as measured by meconium testing was 4.26 (95% CI: 1.34-13.57) times the pooled prevalence of PAE as measured by maternal self-reports. Large variations across the studies in regard to the difference between estimates obtained from maternal self-reports and those obtained from meconium testing were observed.

**Conclusions:**

If maternal self-reports are the sole information source upon which health care professionals rely, a number of infants who were prenatally exposed to alcohol are not being recognized as such. However, further research is needed in order to validate existing biomarkers, as well as discover new biomarkers, for the detection of PAE.

## Background

Prenatal alcohol exposure (PAE) may cause a number of health complications for the mother and the developing fetus, including Fetal Alcohol Spectrum Disorder (FASD). As a “spectrum” disorder, FASD encompasses a broad array of physical defects, cognitive, behavioural, emotional, and adaptive functioning deficits, as well as congenital anomalies, such as malformations and dysplasia of the cardiac, skeletal, renal, ocular, auditory, and other systems [1]. These impairments are likely to have lifelong implications, which result in a significant economic burden for any society. However, the burden of FASD is not measurable by cost alone, the lifelong hardships faced by these children and their families are also of considerable importance.

Identification of infants exposed to alcohol in utero is crucial, as it can lead to close monitoring of his/her development, facilitate early FASD diagnosis, and implement timely interventions, if necessary. Early interventions have long-term benefits for a child with FASD and can potentially reduce the occurrence of secondary disabilities including poor school performance, addictions, mental health problems, sexually deviant behaviour, dependent living, legal issues, and incarceration [2]. Early FASD diagnosis and providing a stable and nurturing environment for the child have been shown to improve outcome and decrease the risk of secondary disabilities by up to fourfold [2,3].

Furthermore, screening of neonates for PAE and early FASD diagnosis can prevent subsequent alcohol-exposed births by providing appropriate interventions, treatment, counselling, and support for birth mothers with unrecognized alcohol dependence and mental health problems [1,4]. Appropriate screening strategies may also facilitate early recognition and intervention for affected siblings.

In order for an infant to be diagnosed with an FASD, PAE needs to be confirmed (with the exception of Fetal Alcohol Syndrome (FAS), which can be diagnosed without PAE confirmation). This can be problematic as maternal self-report data are often under-reported for a variety of reasons (e.g., social desirability bias, recall bias, and/or fear that the child may be taken away). Thus, the detection of PAE in neonates by maternal self-report was shown to be unreliable [5]; thereby necessitating the use of an unbiased biomarker to identify those at risk for FASD.

The use of biological markers has emerged as a practical method for the identification of PAE [6,7]. Currently, there are several measurable biomarkers available for detecting PAE (i.e., fatty acid ethyl esters [FAEE], ethylglucuronide [EtG], ethlysulphate [Ets], and phosphatidylethanol [PEth]) in a range of neonatal matrices (e.g., hair, meconium, blood, placenta, and umbilical cord) [6,8-10]. FAEE in meconium and hair is currently the most commonly used tool to estimate the prevalence of PAE [8]. The validity of the other biomarkers, listed above, remains to be established in neonatal matrices [8].

The detection of FAEE, products of non-oxidative ethanol metabolism, above laboratory cut-points in meconium, has been repeatedly established as a novel biomarker of fetal ethanol exposure in the second and third trimesters of pregnancy [11-19]. Meconium testing for FAEE is a validated method for detecting PAE; it has been shown to have high sensitivity (84.2%) and specificity (83.3%) [11]. Meconium comprises the neonate’s first several bowel movements, identified most commonly by its dark green/black colour and lack of odour. It is generally agreed upon that meconium formation begins at approximately 12 weeks of gestation (i.e., at the end of the first trimester), when fetal swallowing of amniotic fluid is initiated [20,21]; however, some researchers have suggested formation begins even later (up to 20 weeks of gestation) [22-26]. FAEE do not cross the human placenta, causing meconium to serve as a reservoir of fetal chemical exposures during the second and third trimesters of pregnancy. Thus, the presence of FAEE in the meconium is a true reflection of fetal ethanol metabolism [14]. Cumulative meconium FAEE concentrations exceeding 2.0 n▪mol/g, the internationally accepted cut-point [13], may be indicative of seven or more drinks per week or five or more drinks per occasion (i.e., “binge” drinking) [15,27].

The purpose of the current study was to compare the prevalence of PAE, as reported via maternal self-reports, with the results of meconium testing (the most commonly used laboratory screening method for estimating the prevalence of PAE, with proven accuracy), and to quantify the disparity between these two methods.

## Methods

The systematic literature review and meta-analyses were conducted and reported according to the standards set out in Preferred Reporting Items for Systematic Reviews and Meta-Analyses (PRISMA; http://www.prisma-statement.org/) [28].

### Ethics statement

The current study utilized secondary data reported on the aggregate level, which is readily available in the literature; therefore, it was not necessary to obtain research ethics approval.

### Literature search

A literature search was performed to identify published studies that have estimated the prevalence of PAE at any level and at any point of time during pregnancy using maternal self-reports and meconium testing.

The search was conducted in multiple electronic bibliographic databases, including: Ovid MEDLINE, PubMed, EMBASE, Web of Science (including Science Citation Index, Social Sciences Citation Index, Arts and Humanities Citation Index), BIOSIS, PsycINFO, Social Work Abstracts, Scopus, and Google Scholar. The search was conducted using multiple combinations of the following key words: 1) alcohol, ethanol, FASD, FAS, pFAS, ARND, ARBD, PAE; 2) consum*, drink*; 3) maternal, mother, primigravida, wom*n; 4) pregnan*, prenatal; 5) biomarker, fatty acid ethyl ester, meconium (analysis), self-report; and 6) prevalence, frequenc*, occurrence.

In addition, manual reviews of the content pages of the major epidemiological journals were conducted, as well as citations in the relevant articles. The search was not limited geographically or by language of publication and was conducted on studies published before January 2013, inclusively.

### Eligibility criteria

Articles were retained if they met the following eligibility criteria: i) consisted of original, quantitative research on human participants published in a peer-reviewed journal; and ii) obtained a measurement of maternal alcohol use during pregnancy via self-reports (e.g., interview, questionnaire, etc.) in combination with meconium testing for FAEE with a pre-defined cumulative cut-point (this criterion was used as a quality control measure and was necessary for comparability purposes).

It must be recognized that because of the latter inclusion criterion, the pooled estimates obtained for maternal self-reports and for meconium testing cannot be used to make any inferences on their own; however, they can be used to quantify the pooled difference between these two methods of ascertainment (the purpose of the current study).

Studies were first screened using titles and abstracts; of those retained, the eligibility criteria were applied, and preference was given to full-text peer reviewed journal publications in all cases.

### Data extraction

One member of the study team independently extracted the data from the available articles, while a second investigator checked table entries for accuracy against the original articles. All discrepancies were reconciled by team discussion. The following data were extracted from each article, wherever available: the country in which the study was conducted (as well as the specific provinces, territories or states, if such information was available), the year(s) of the study (i.e., the year(s) in which data collection took place), the sample size, maternal age, the prevalence of PAE via self-reported data, the assessment tool used to obtain self-reported alcohol use, the trimester during which alcohol was consumed, the frequency of alcohol use, the prevalence of PAE via meconium testing, and the cumulative FAEE cut-point used.

### Statistical analysis

To combine the prevalence estimates of PAE as measured by maternal self-reports and by meconium testing, meta-analyses were performed using the Mantel-Haenszel method, assuming a random-effects model [29]. Before performing the meta-analyses, prevalence estimates were transformed using a double arcsine transformation so that the data followed a normal distribution (an assumption needed when statistically combining estimates) [30]. Heterogeneity between studies was assessed using the Cochrane Q-test and the I^2^ statistic [31,32]. Results of the meta-analyses were displayed using Forest plots. Publication bias was assessed using a ranked correlation test [33], and by employing a weighted regression test [34]; however, given that the prevalence estimates are unlikely to be affected by publication bias [35], if present, publication bias would not be adjusted for.

To test for a difference in the prevalence estimates of PAE as measured by maternal self-reports and those obtained by meconium testing, a random effects meta-regression (using a logit regression [using log odds transformed prevalence estimates]) was performed. The correlation between estimates from the same study was corrected for in the meta-regression model. The prevalence estimates as reported by Manich and colleagues [36] were excluded from the meta-regression, as the standard error of the log odds transformed prevalence estimates of 0 are undefined.

All statistics were performed using STATA version 11.2.

## Results

### Characteristics of the included studies

Initially, the electronic search yielded a total of 839 publications regarding the prevalence of PAE, measured using maternal self-reports and meconium testing, when using the key words specified above (there were no articles identified through other sources - i.e., the manual search). After removing 501 duplicate articles, a total of 338 articles were screened using titles and abstracts. Fifty-six full-text articles were retrieved for further consideration, 47 of which were subsequently excluded. A total of 9 articles were retained and selected for data extraction.

**Figure 1:**
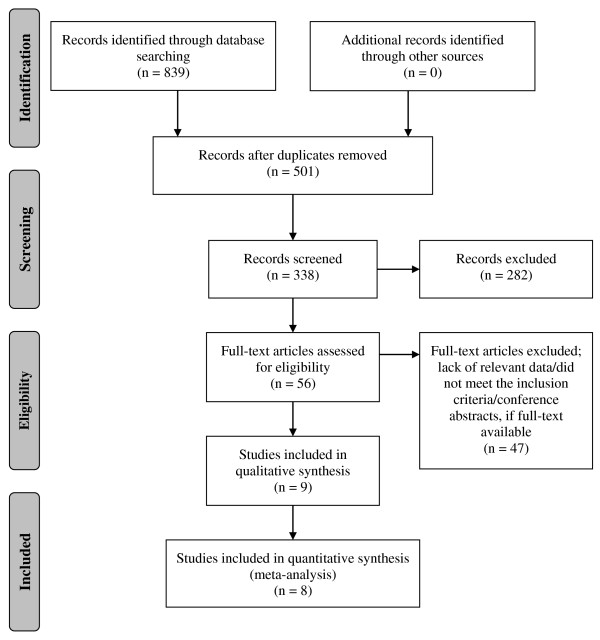
Flow diagram depicting the search strategy employed.

A schematic diagram of the search strategy is depicted in Figure 1.

Nine studies on the prevalence of PAE, obtained via maternal self-reports and meconium testing (Hutson et al. [37] and Magri et al. [38,39] were published in iteration, and Derauf et al. [40,41] are dual publications), were identified from the literature. Data on the prevalence of PAE, obtained via maternal self-reports and meconium testing, were available from only six countries: Canada [15,42], Germany [43], Italy [44,45], Spain [36,44,46], Uruguay [37-39], and the United States [40,41].

Please see Table 1 for the characteristics of the studies available on the prevalence on PAE obtained via maternal self-reports and meconium testing.

**Table 1:** Studies that reported the prevalence of prenatal alcohol exposure using maternal self-reports and meconium testing

**Country, province/territory or state (if available)**	**Reference**	**Year(s) of study**	**Sample size**	**Setting**	**Maternal age (mean; years)**	**Tool used to obtain self-reported data**	**Trimester when alcohol was consumed, number of mothers and percent of self-reported drinkers (if available)**	**Cumulative FAEE cut-point**	**Prevalence of prenatal alcohol exposure**				**Increase in sensitivity (meconium testing versus self-reports)**^**a**^
									**Prevalence via self-reports**		**Prevalence via meconium testing**		
									**Number of mothers**	**Percentage of mothers**	**Number of mothers**	**Percentage of mothers**	
Canada, Ontario	Gareri et al. [15]	2004-05	682	Regional birthing hospitals	n/a	Questionnaire	n/a	2 n▪mol/g	5	0.5%	17	2.5%	5 times
Canada, Ontario	Goh et al. [42]	2006-07	50	High-risk obstetric unit	n/a	Medical record	n/a	2 n▪mol/g	1	2.0%	15	30.0%	15 times
Germany, Erlangen	Bakdash et al. [43]	n/a	602	Department of Obstetrics and Gynecology	n/a	Questionnaire, with CAGE [47]	n/a	500 n▪g/g (~2 n▪mol/g)	1	0.2%	43	7.1%	43 times
Italy, Emilia	Pichini et al. [44]^b^	n/a	96	Neonatal Intensive Care Unit	28.4	Questionnaire	n/a	2 n▪mol/g	3	3.1%	8	8.3%	2.7 times
Italy	Pichini et al. [45]	n/a	607	Neonatal wards of public hospitals	31	Questionnaire, with AUDIT [48]	All three trimesters	2 n▪mol/g	65^c^	10.7%	48	7.9%	0.7 times
Spain, Barcelona	Garcia-Algar et al. [46]	n/a	353	Hospital (low SES)	29	n/a	n/a	2 n▪mol/g	48	13.6%	159	45.0%	3.3 times
Spain, Barcelona	Manich et al. [36]	n/a	62	Neonatal hospital	n/a	Questionnaire	n/a	2 n▪mol/g	0	0%	10	16.1%	16.1 times
Spain, Barcelona	Pichini et al. [44]^b^	n/a	81	Neonatal Intensive Care Unit	28.3	Questionnaire	n/a	2 n▪mol/g	4	4.9%	34	42.0%	8.5 times
Uruguay, Montevideo	Hutson et al. [37]; Magri et al. [38,39]^d^	2005	900	Public Hospitals	25.4	Questionnaire, with CAGE [47]	n/a	2 n▪mol/g	331	36.8%	362	43.5%	1.2 times
USA, Hawaii	Derauf et al. [40,41]^e^	1999	422	Urban regional perinatal center	29 (median)	Medical record	1^st^ trimester: 5 (21.7%); 1^st^ & 2^nd^ trimester: 2 (8.7%); All three trimesters: 4 (17.4%); Not documented: 12 (52.2%)	50 n▪g/g	18^f^	4.3%	72	17.1%	4.0 times

AUDIT: Alcohol Use Disorders Identification Test; FAEE: fatty acid ethyl esters; n/a: not available; SES: socio-economic status.

^a^Levels of increased sensitivity may not total percentage figures obtained via meconium testing divided by percentage figures obtained via self-reports due to rounding errors.

^b^Pichini et al. [44] reported results for two study sites (Italy and Spain).

^c^Reported “daily” consumption.

^d^Hutson et al. [37] and Magri et al. [38,39] were published in iteration.

^e^Derauf et al. [40,41] are dual publication.

^f^Excludes 5 women who reported alcohol use during the first trimester only.

With respect to meconium testing, eight of the identified studies used the internationally accepted cumulative FAEE cut-point of 2 n▪mol/g, while Derauf et al. [40,41] used 50 n▪g/g, which is approximately 10 times lower.

**Figure 2:**
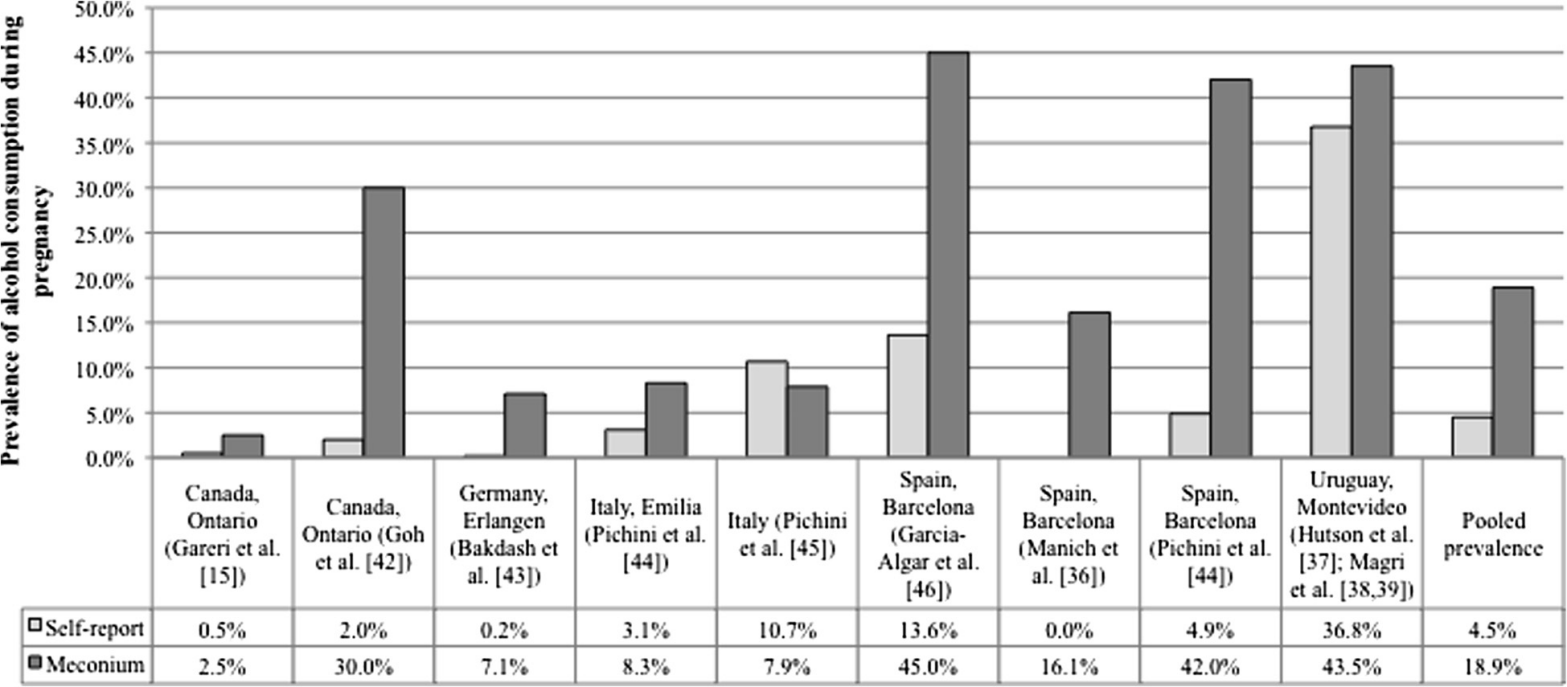
The prevalence of prenatal alcohol exposure obtained using maternal self-reports versus meconium testing and the pooled prevalence estimate.

Based on the self-reported data, the prevalence of PAE ranged from 0% (in Spain [36]) to 36.8% (in Uruguay [37-39]). In comparison, based on the meconium testing data, the prevalence of PAE ranged from 2.5% (in Canada [15]) to 45.0% (in Spain [46]; Table 1). The prevalence of PAE, obtained via meconium testing, ranged from 1.2 times (in Uruguay [37-39]) to 43 times higher (in Germany [43]) than that obtained via maternal self-reports (Table 1 and Figure 2).

**Figure 3:**
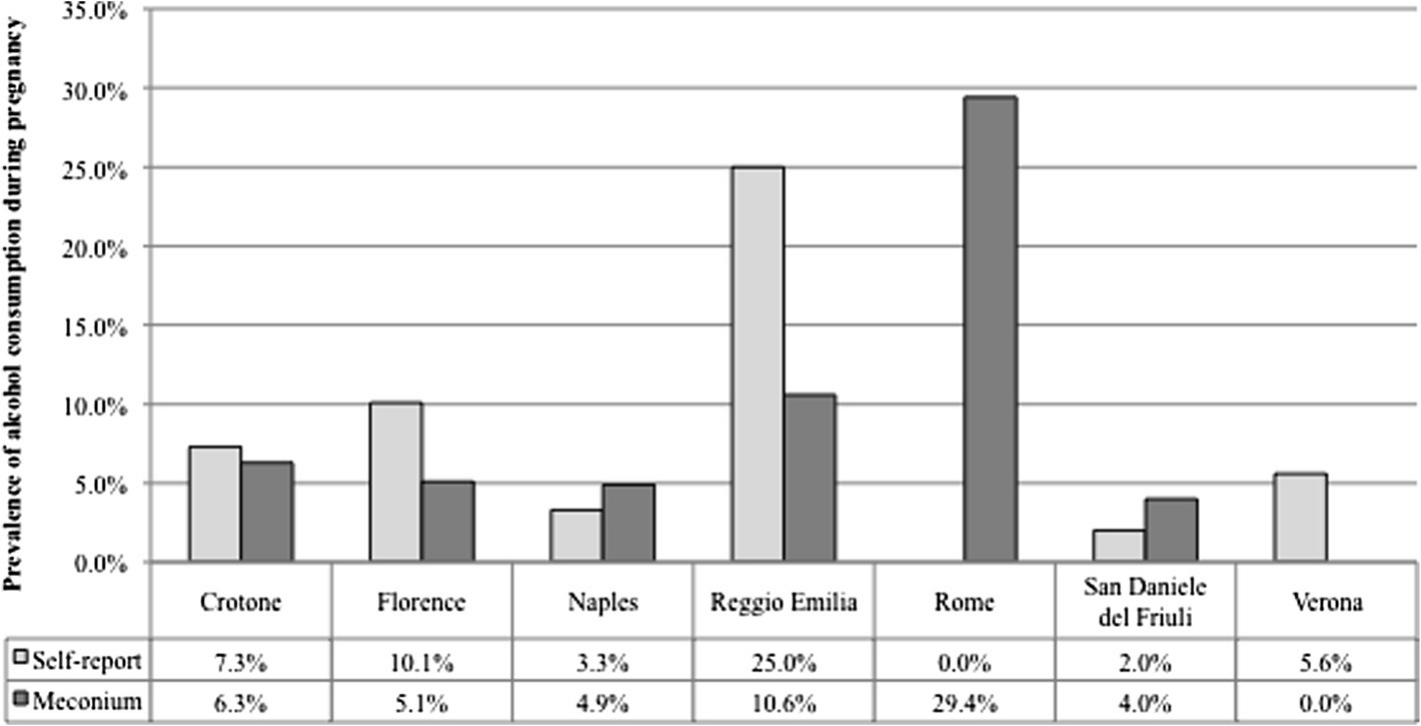
**The prevalence of prenatal alcohol exposure obtained using maternal self-reports versus meconium testing across seven sites in Italy.** Footnote. Source: Pichini et al. [45].

In one of the studies from Italy [45], which investigated the prevalence of PAE across seven sites, four of the sites reported a higher prevalence using maternal self-reports compared to meconium testing, while three sites reported a higher prevalence using meconium testing, compared to maternal self-reports (Figure 3). As a result, in Pichini et al. [45] the pooled prevalence of PAE based on maternal self-reports across all seven sites was 1.4 times higher than the prevalence obtained via meconium testing.

### Pooled prevalence of PAE obtained via maternal self-reports versus meconium testing

Eight studies were included in the meta-analysis. The study by Derauf et al. [40,41] was excluded because the cumulative FAEE cut-point used in this study was about 10 times lower than the cumulative FAEE cut-points used in the other studies (2 n▪mol/g; see Table 1).

**Figure 4:**
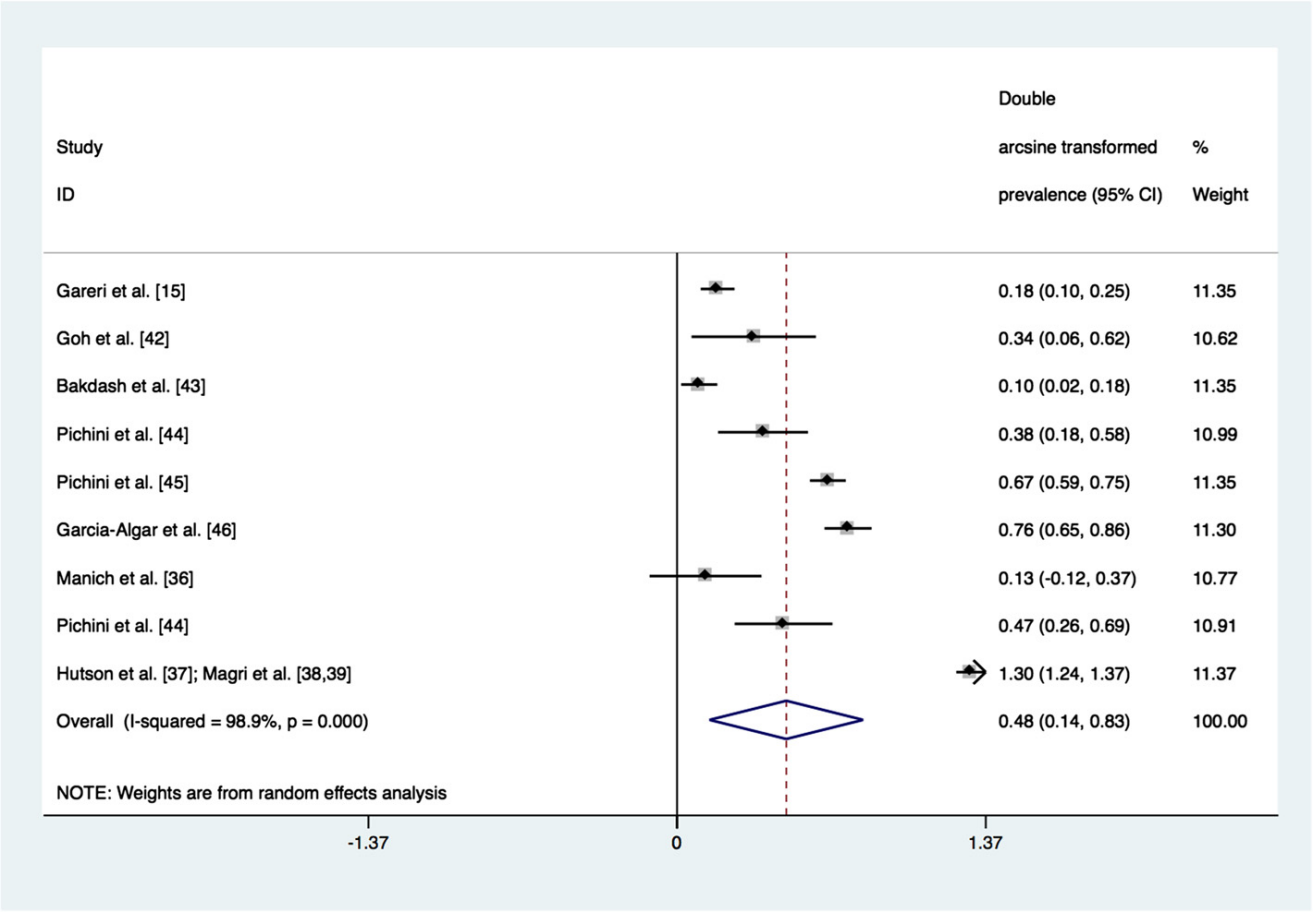
**Forest plot of the prevalence of prenatal alcohol exposure as measured by maternal self-reports**^**a**^**.** Footnote. CI: confidence interval. ^a^ The size of the box around the point estimate is representative of the weight of the estimate used in calculating the aggregated point estimate.

**Figure 5:**
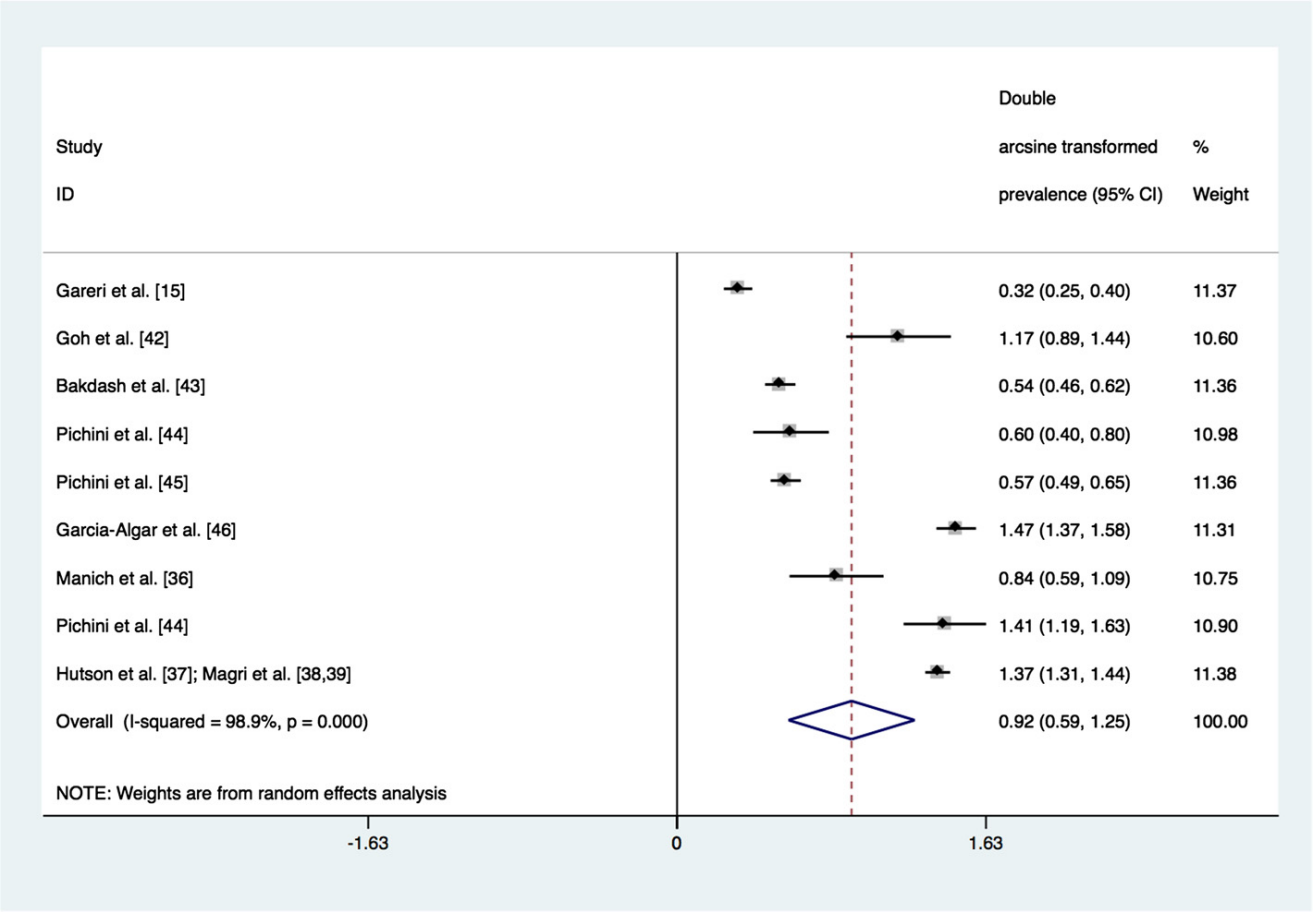
**Forest plot of the prevalence of prenatal alcohol exposure as measured by meconium testing**^**a**^**.** Footnote. CI: confidence interval. ^a^ The size of the box around the point estimate is representative of the weight of the estimate used in calculating the aggregated point estimate.

**Figure 6:**
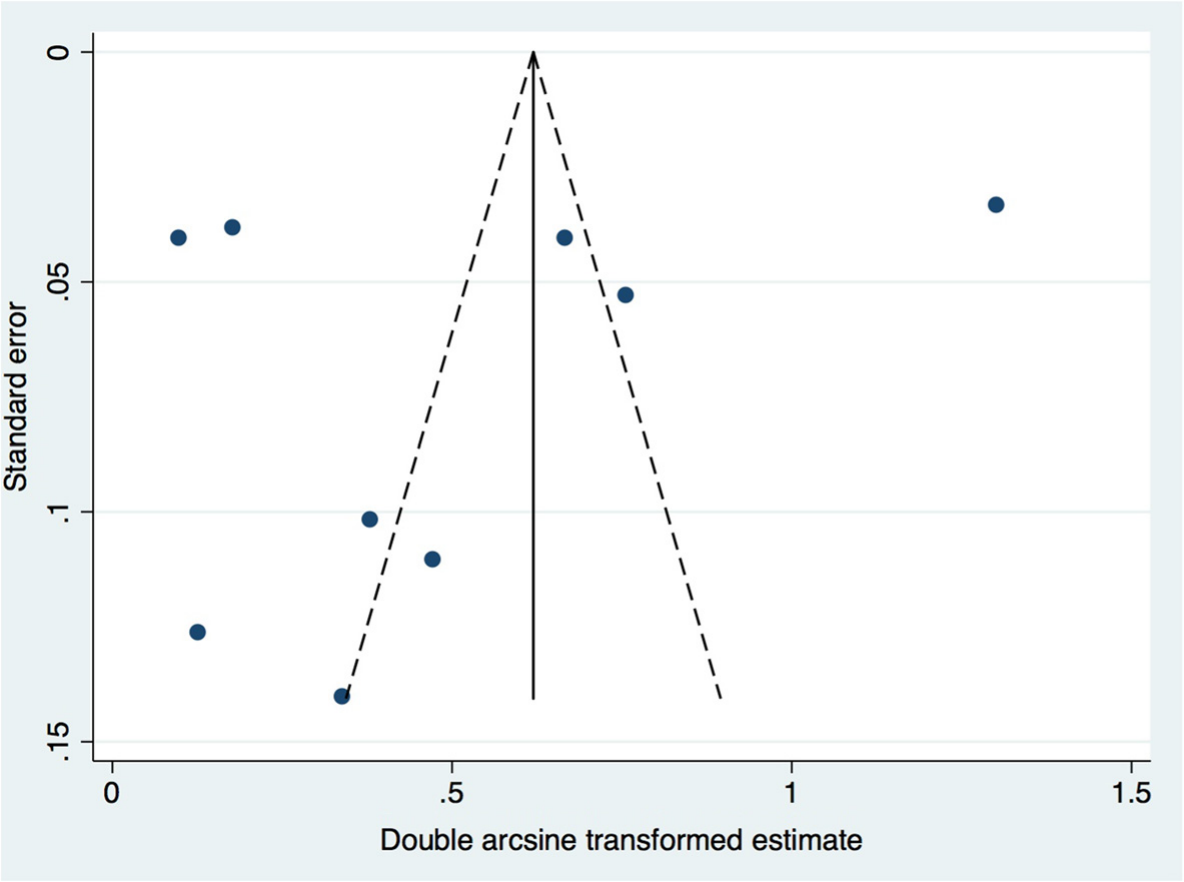
Funnel plot of the prevalence of prenatal alcohol exposure as measured by maternal self-reports.

**Figure 7:**
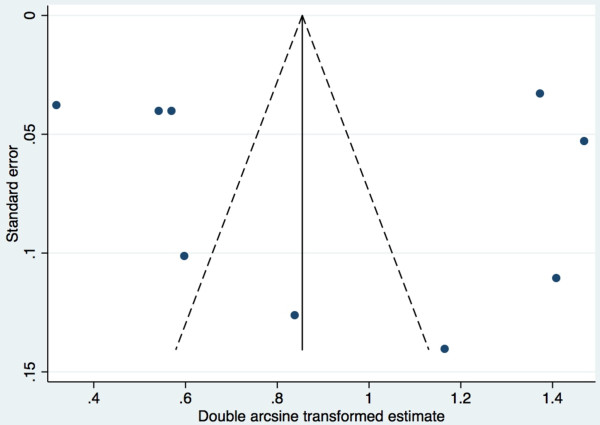
Funnel plot of the prevalence of prenatal alcohol exposure as measured by meconium testing.

The meta-analysis revealed that the pooled prevalence of PAE obtained by maternal self-reports was 4.5% (95% confidence interval (CI): 0.1% to 15.1%; see Figure 4 for Forest plot). Tests demonstrate that heterogeneity in these estimates was present (Q_(8)_ = 751.70, *p* = 0.000; I^2^ = 98.9%, *p* = 0.000). The pooled prevalence of PAE obtained by meconium testing was 18.9% (95% CI: 8.4% to 33.9%; see Figure 5 for Forest plot). Tests demonstrate that heterogeneity in these estimates was also present (Q_(8)_ = 714.67, *p* = 0.000; I^2^ = 98.9%, *p* = 0.000). Begg’s rank correlation test (p = 0.602 and p = 0.602 for the meta-analysis of estimates of PAE as obtained from maternal self-reports and meconium testing, respectively) [33] and Egger weighted regression test (p = 0.493 and p = 0.807 for the meta-analysis of estimates of PAE as obtained from maternal self-reports and meconium testing, respectively) [34] both indicated that publication bias was not present for these meta-analyses (see Figure 6 and Figure 7 for Funnel plots). Based on the estimates presented above, meconium testing resulted in values 14.4% higher than maternal self-reports.

The meta-regression using log odds transformed prevalence estimates indicated that the prevalence of PAE as measured by meconium testing was 4.26 (95% CI: 1.34 to 13.57) times the prevalence of PAE as measured by maternal self-reports (*p* = 0.021; see Table 2).

**Table 2:** Results of the meta-regression testing for a difference in the prevalence estimates of prenatal alcohol exposure as measured by maternal self-reports and those obtained by meconium testing

**Model**	**Measure**	**Point estimate**	**Lower 95% confidence interval**	**Upper 95% confidence interval**	**p-value**
Log-odds model 1^a^	Meconium testing (as compared to maternal self-reports)	4.41	0.82	23.76	0.08
Log-odds model 2^b^	Meconium testing (as compared to maternal self-reports)	4.26	1.34	13.57	0.02

^a^Not controlling for between study differences in measurement.

^b^Controlling for between study differences in measurement.

## Discussion

The prevalence estimates of PAE will be, on average, four times higher when using meconium testing compared to the prevalence estimates obtained using maternal self-reports; however, it is not currently feasible to use meconium testing to screen every newborn or to implement a universal meconium testing screening program. There are also legal and ethical issues surrounding meconium testing and its use as a universal screening tool (see Dickens [49]), which need to be resolved before implementation of such a screening program takes place. Thus, a maternal self-report method is the most widely used at the present time.

The current study has limitations due to the lack of consistency across studies with respect to maternal self-report data. For instance, the studies used different instruments/methods to obtain the self-reported data, each with its own sensitivity and specificity. Furthermore, only one study [45] had provided a breakdown by frequency of alcohol use: “daily”, “weekly”, and “monthly” (however, the amount consumed was not reported). In addition, the majority of studies did not specify the time period captured by the exposure assessment, and as a result, it cannot be said definitively that the maternal self-reported data is reflective of the same time period as the meconium testing data (which detects alcohol use in the second and third trimesters only).

Mecounium testing also has several limitations. Due to the inability of meconium testing to ascertain PAE during the first trimester and to “detect” if pregnant women consumed less than seven drinks per week and/or did not practice “binge” drinking (five or more drinks per occasion) during the second and third trimesters of pregnancy, the prevalence of PAE obtained using this method is likely to be an underestimate and, thus, the pooled difference reported in this paper is likely to be underestimated. However, a positive meconium test (i.e., above the cumulative FAEE cut-point) indicated heavy drinking in the second and third trimesters. In countries where the majority of women are aware of the adverse effects of alcohol consumption on the developing fetus (e.g., Australia and Canada [50,51]), a positive meconium test likely indicates continuous drinking during pregnancy and thus, alcohol dependence; therefore, meconium testing can be said to identify the most at-risk group of neonates.

Additionally, there are variations in the laboratories’ methods for testing meconium for the presence of FAEE (e.g., in the extraction methods used, and in the analytical strategies employed), which affect the PAE prevalence estimates obtained [52]. Furthermore, the association between level of alcohol consumption and the level of FAEE is difficult to ascertain due to individual differences of both the mother and the fetus [52].

Given the limitations of meconium testing, one should err on the side of caution when screening for PAE, as there are potentially serious consequences of both false positive and false negative results. This is especially important when a “positive” test is used for any other purpose than to identify individuals at-risk and in need of intervention, such as when PAE is used as an alternative explanation for poor birth outcomes in medical malpractice claims. A “positive” meconium test result could also lead to increased contact with child protective services in some countries. Accordingly, there is a need for research that can lead to improvements in the sensitivity and specificity of meconium testing so that a universal standard can be definitively established.

The results of this study also reveal that existing estimates of the prevalence of PAE (regardless of the method of detection) vary across countries, as well as within countries. Furthermore, large variations in the estimates of PAE as measured by maternal self-reports were observed when compared to estimates of PAE obtained from meconium testing. Between country variations in the prevalence of PAE likely stem from differences in maternal drinking behaviours, as well as from political, ideological, cultural, and legal differences between countries. As discussed in a recent study by Drabble and colleagues [53], countries vary considerably in the quality and type of public messaging in terms of levels of political attention, responsiveness of governments, and the information relayed to the public regarding alcohol and pregnancy. For countries in which there is heightened political attention, responsiveness, and public messaging regarding alcohol use during pregnancy, there is an increased understanding of the risk associated with alcohol consumption and pregnancy, and consequently, a lower observed prevalence of PAE.

Differences in ideological and cultural conditions between countries can also influence both the prevalence of alcohol use during pregnancy and the accuracy of maternal self-reports [53]. For example, the social acceptability of alcohol use among women influences the stigmatization of women who do consume alcohol, especially during pregnancy [53]. Thus, in those countries where alcohol consumption by women is not socially acceptable, women are less likely to report their use of alcohol during pregnancy. This could explain the results reported for Uruguay [37-39], where maternal self-reports were not that different from the results of the meconium testing, and could explain the results from some study sites in Italy [45], where maternal self-reports yielded higher prevalence estimates compared to meconium testing. It is possible that in Uruguay and in Italy alcohol consumption by women is less stigmatized when compared to other countries, and thus, women are more likely to accurately report their alcohol consumptions during pregnancy (Magri R 2013, personal communication, May 11). The results from Uruguay and Italy may be explained further by the potential that “low/moderate” levels of alcohol consumption were not “detected” by meconium testing, whereas these levels can be measured by maternal self-reports. Furthermore, a lack of public awareness may exist in Uruguay and in Italy of the deleterious effects of alcohol on a developing fetus, and as a consequence, women in these countries may be more willing to reveal alcohol use during pregnancy [53].

Finally, laws concerning alcohol use during pregnancy may impact variations in self-reported PAE. In Canada, for example, women are not held legally responsible for prenatal injuries, as holding a woman legally responsible in these circumstances is considered to a violation of her fundamental rights [49]. Therefore, an absence of legal consequences of PAE may cause women to be more willing to disclose their alcohol use during pregnancy.

## Conclusions

Based on the results of this study, health care professionals should be aware of the under-reporting of PAE by maternal self-reports. If maternal self-reports are not adjusted for under-reporting, a number of infants prenatally exposed to alcohol will not being recognized as such. Although meconium testing provides more accurate data, this paper advocates for the use of meconium testing in addition to maternal self-reports when testing for PAE. However, given that meconium testing cannot determine exposure in the first trimester, further research is needed in order to discover new and validate existing biomarkers for the detection of PAE. As indicated above, there are a number of biomarkers available for detecting PAE in a range of neonatal matrices. These biomarkers need to be further explored and the accuracy (i.e., sensitivity and specificity) of the methods employed needs to be established; however, some preliminary results appear promising (see for example, Bakhireva et al. [54], Matlow et al. [55], and Shukla et al. [56]).

## Abbreviations

FASD: Fetal alcohol spectrum disorder; PAE: Prenatal alcohol exposure.

## Competing interests

The authors declare that they have no competing interests.

## Authors’ contributions

SL led the conception and design of the study, acquired the data, analyzed and interpreted the data, wrote the first draft of the manuscript, and revised the manuscript. KS analyzed and interpreted the data, and revised the manuscript critically for important intellectual content. JR and GK contributed to data interpretation, and have revised the manuscript critically for important intellectual content. SP contributed to the conception and design of the study, supervised the analysis and interpretation of the data, and participated in drafting and revising the manuscript. All authors have provided final approval of the version to be published, and have agreed to be accountable for all aspects of the work in ensuring that questions related to the accuracy or integrity of any part of the work are appropriately investigated and resolved.

## Pre-publication history

The pre-publication history for this paper can be accessed here:

http://www.biomedcentral.com/1471-2393/14/127/prepub
